# MicroRNA, Proteins, and Metabolites as Novel Biomarkers for Prediabetes, Diabetes, and Related Complications

**DOI:** 10.3389/fendo.2018.00180

**Published:** 2018-04-23

**Authors:** Suniti Vaishya, Rucha D. Sarwade, Vasudevan Seshadri

**Affiliations:** ^1^National Centre for Cell Science, Pune, India; ^2^Department of Biotechnology, Savitribai Phule Pune University, Pune, India

**Keywords:** microRNA, proteins, metabolites, type 2 diabetes mellitus, biomarker

## Abstract

Type 2 diabetes mellitus (T2DM) is no more a lifestyle disease of developed countries. It has emerged as a major health problem worldwide including developing countries. However, how diabetes could be detected at an early stage (prediabetes) to prevent the progression of disease is still unclear. Currently used biomarkers like glycated hemoglobin and assessment of blood glucose level have their own limitations. These classical markers can be detected when the disease is already established. Prognosis of disease at early stages and prediction of population at a higher risk require identification of specific markers that are sensitive enough to be detected at early stages of disease. Biomarkers which could predict the risk of disease in people will be useful for developing preventive/proactive therapies to those individuals who are at a higher risk of developing the disease. Recent studies suggested that the expression of biomolecules including microRNAs, proteins, and metabolites specifically change during the progression of T2DM and related complications, suggestive of disease pathology. Owing to their omnipresence in body fluids and their association with onset, progression, and pathogenesis of T2DM, these biomolecules can be potential biomarker for prognosis, diagnosis, and management of disease. In this article, we summarize biomolecules that could be potential biomarkers and their signature changes associated with T2DM and related complications during disease pathogenesis.

## Introduction

Type 2 diabetes mellitus (T2DM) is a metabolic disorder which is the cause of serious concern worldwide. According to the International Diabetes Federation report, global burden of diabetes affected population will be increased to ~592 million by the year 2035 (1). Developing countries like India will have ~109 million of affected people, making diabetes no more a developed world disease. Characterized by hyperglycemia, T2DM is a group of metabolic disorders resulting from defects in insulin secretion, action or both (2). After the onset of disease, T2DM further leads to macrovascular, microvascular, and neurological complications. The clinical manifestation of a complex metabolic disease like T2DM is delayed by years, thereby restricting its timely diagnosis. It has been demonstrated that there is a significant relative risk reduction of cardiovascular Diseases (CVDs) and all-cause mortality in individuals undergoing regular medical examination emphasizing the importance of early diagnosis of the disease (3). Thus, the identification of high-risk individuals even before the prediabetes stage, when the beta cells are relatively intact, is of paramount importance for effective intervention, preventing progression to overt disease.

Despite several advances in understanding of disease establishment, progression, and related pharmacotherapy, there has been a sustained increase in diabetes-affected population. The availability of effective interventions for prevention and delaying the onset of T2DM has created urgency for the early identification of individuals at risk. Besides, as larger proportion of affected population now comes from developing countries, cost-effectiveness and affordability of diagnostic tests are also an issue. The practical difficulty in procuring samples to test blood glucose and serum insulin levels, the intra-individual variability in blood glucose levels (4), the high-risk familial genes adding no value in predicting the onset of the disease (5), and the fact that only 20% of the obese individuals are actually at a risk of developing full-blown T2DM (6) limits the use of existing biomarkers.

A biomarker is a biomolecule/biological state that could be used for the prognosis, diagnosis, and follow-up of the pathological state of a disease. These could also be used to assess the severity of disease. There are different kinds of biomarkers [including biomolecules like proteins, microRNAs (miRNA), etc.] that are being used for the diagnosis of many diseases like T2DM, cancer, neurological diseases, etc. (7, 8). Pathophysiology of T2DM is substantially contributed by adipocyte signaling (9), inflammation (10), endothelial dysfunction (11), iron overload (12), incretin abnormalities (13), and inconsistencies in circadian systems (14, 15). Biomarkers that are designed taking into account these parameters would help improve screening approaches for T2DM.

## Current Understanding of Diagnostic Markers for T2DM

The current diagnostic thresholds for T2DM according to Expert Committee on the Diagnosis and Classification of Diabetes are fasting plasma glucose (FPG) of ≥126 mg/dl (7.0 mmol/l) and 2-h post-prandial plasma glucose of ≥200 mg/dl (11.1 mmol/l) and HbA1c of ≥6.5% (47.5 mmol/mol). These criteria are largely based on an association of cross-sectional glycemic and HbA1c levels, respectively, with diabetic retinopathy (2, 16). Prediabetes is an intermediate group of individuals whose plasma glucose levels range between normoglycemia and diabetes. The fasting glucose concentration of 110–125 mg/dl (6.1–6.9 mmol/l) by World Health Organization (WHO) and 100–125 mg/dl (5.6–6.9 mmol/l) by American diabetes association (ADA) [also termed impaired fasting glucose (IFG)], and ADA and WHO recommended 2-h glucose concentration cutoffs of 140–199 mg/dl (7.8–11.0 mmol/l) [also termed impaired glucose tolerance (IGT)] specifically define the condition of prediabetes and their long-term outcomes (16). People with IFG, IGT (2 h after meal), or abnormal HbA1c values have a higher risk of developing T2DM and associated pathophysiology. Several clinical characteristics have been associated with T2DM (17). Apart from blood glucose level and HbA1c, other parameters like levels of blood cholesterol, lipoproteins, C-peptide, blood pressure, etc. are also taken into account to predict T2DM (18). However, except for blood glucose assessment and HbA1c, all other parameters are not specific to T2DM (18). Moreover, hyperglycemia assessed by fasting glucose and 2-h blood glucose estimation only gives an idea of the glucose level at a single time point. Hence, testing them more than once is required. Also, the complexity of clinical condition cannot be fully described by blood glucose level. HbA1c is a more reliable measurement than fasting glucose level as it indicates the average levels of plasma glucose over several weeks and is better correlated with chronic complications. HbA1c assessment does not require fasting and has a greater pre-analytical stability than plasma glucose. Hyperglycemia is the main biochemical phenomenon associated with diabetes which further leads to elevation in glycated proteins (HbA1c). However, there is a time gap between hyperglycemia and rise in glycated hemoglobin level, resulting in delayed diagnosis of the onset of diabetes using HbA1c levels. Standardization of HbA1c assay is required, whereas glucose assay is standardized and easier to implement. Moreover, most of these classical biomarkers are useful only after the establishment of disease and fails to predict disease at a prediabetic condition. Besides, classical markers are inconclusive to predict the pathogenesis of T2DM which eventually leads to severe complications including chronic heart disease, diabetic nephropathy (DN), retinopathy, etc. Thus, novel, more specific, noninvasive, stage-related biomarkers that are accurate in the diagnosis of initiation and progression of T2DM are needed.

## Emerging Role of miRNAs as Biomarkers in Type 2 Diabetes and Related Complications

MicroRNAs are small, 20–25 nt long noncoding RNA molecules which normally binds to the 3′ end of its target mRNAs to inhibit its translation, eventually leading to a reduced gene expression (19). miRNAs can target multiple genes and are involved in the regulation of multiple functions in cells. It has been estimated that miRNA target site is conserved in the 3′ UTR of more than 60% of all mammalian mRNAs; thus, miRNAs are likely to be important regulators in the cell (20). Owing to their stability and presence in various body fluids, miRNAs emerged as potential biomarkers for T2DM and related complications. Besides, the differential expression of miRNAs in various tissues has been reported in T2DM and related complications (Table 1). It has been suggested that miR-103 and miR-143 may regulate the subcutaneous adipose tissue and the development of T2DM in mice. miR-103 may also be involved in the regulation of adipose and the control of glucose metabolism in humans (21). Platelet-derived miR-103 was found to negatively regulate the expression of secreted frizzled-related protein 4, which is a potential risk biomarker for the onset of diabetes mellitus (prediabetes). miR-103 was downregulated in individuals with prediabetes (22). The expression of various miRNAs is altered in patients with diabetes-related complications including microvascular complications (23). It has been suggested that the expression of miRNAs in different tissues and body fluids also reflects disease pathology. For instance, the expression of five miRNAs miR-661, miR-571, miR-770-5p, miR-892b, and miR-1303 were increased in T2DM (23). miR-126, miR-320, and miR-503 have also been shown to be involved in diabetic cardiovascular disease and endothelial function (24–27). miR-320 expression was also elevated in diabetic rat models (26). Increased levels of miR-18a and decreased levels of miR-34c in peripheral blood mononuclear cells are important markers of chronic stress response and may play a role in T2DM-risk assessment (28). miR-375, an miRNA expressed in pancreas reduces the level of PDK1 kinase, leading to a decreased glucose-stimulated insulin gene expression. The role of miR-375 seems to be conserved across species including humans and mice. miR-375 plays a role in the process of re-differentiating mature human beta cells *in vitro* and hence can be useful for cell replacement therapy in diabetes. An increased expression of miR-375 in expanded islet cells dampens the PDK1–AKT pathway, as well as GSK3-signaling pathways leading to the regeneration of insulin-producing beta cells (29). Besides, the overexpression of miR-375 suppresses glucose-stimulated insulin secretion by downregulating the expression of myotrophin (30, 31). Defective insulin secretion is observed in many T2DM instances, and miR-126 seems to play an important role in this process and exhibits a negative relationship with T1DM and T2DM (32, 33). In relation to T2DM, a unique circulatory miRNA signature has been identified (34). The levels of four miRNAs miR-126, miR-15a, miR-29b, and miR-223 decrease while miR-28-3p level increases in case of T2DM. These miRNAs are significantly modulated even before the manifestation of the disease, making these small molecules valuable as a prognostic marker for the prediction of T2DM (34). Imbalance in the exocytotic machinery components leads to impaired insulin secretion by the pancreatic β-cells, resulting in T2DM. Studies have shown the significance of miRNA in the regulation of glucose-stimulated insulin translation, secretion, and exocytosis by pancreatic cells (35–37). Increased levels of miR-335 lead to impaired insulin secretion (38). miR-196a was shown to be regulating the insulin biosynthesis, and its role was suggested to be important during early embryonic development (36). Interestingly, it was shown that the restoration of specific miRNAs can attenuate the progression of disease in animal models. For instance, the rescue of a reduced level of miR-181b in epithelial cells of adipose tissue in a mouse model of obesity leads to an improvement in glucose homeostasis and insulin sensitivity (39). Silent mating type information regulation 2 homolog 1 (Sirt1) is involved in neuroprotection and wound healing. Sirt1 regulates the expression of miR-182 which further overcomes the detrimental effects of hyperglycemia by decreasing the expression of NOX4, leading to corneal nerve regeneration (40). Progression of diabetes leads to complications like diabetic kidney disease (DKD). In comparison to diabetic individuals, patients with DKD were found to be differentially expressing (>2-fold) 496 urinary exosome-derived miRNA species. Four of these were further validated, and it was reported that miR-362-3p, miR-877-3p, and miR-150-5p were upregulated, while miR-15a-5p was downregulated. These miRNAs might be involved in the regulation of DKD through p53, mTOR, and AMPK pathways (41). Similarly, various miRNAs were found to be involved in endothelial function and diabetic cardiovascular diseases (CVD) (42, 43). For instance, miR-126 exhibits a lower expression in coronary artery disease and myocardial infarction patients compared with healthy controls. It was suggested that miR-126 regulates endothelial cells by targeting sprout-related protein *via* Ras/ERK/VEGF and PI3K/Akt/eNOS pathways (43).

**Table 1:** MicroRNAs (miRNAs) associated with type 2 diabetes mellitus (T2DM) and associated complications.

S. No.		miRNA	Target	Expression level	Reference
1.	Obesity and T2DM	miR-124a	Mtpn, Foxa2, Flot2, Akt3, Sirt1, and NeuroD1	Up	(22)
2.		miR-101		Up	(44)
3.		miR-802		Up	(44)
4.		miR-96	Noc2	Down	(35)
5.		miR-103	SFRP4	Up	(45)
6.		miR-375	Mtpn, PDK1	Up/down	(44)
7.		miR-23a	SMAD4	Up	(21)
8.		miR-132	NF-kappa B	Down	(46)
9.		miR-34a	SIRT1	Down	(46)
10.		miR-145	ADAM17	Down	(21)
11.		miR-221	CAV-1	Up	(46)
12.		miR-144	IRS-1	Up	(21)
13.		miR-146a	TRAF6	Up	(21)
14.		miR-29	Spry1, AKT3	Up	(21)
15.		miR-34a	FGFR1, BetaKL	Up	(21)
16.		miR-15a	UP2	Down	(34)
17.		miR-126	IRS-1	Down	(34, 46)
18.		miR-29b	DNMT1	Down	(34)
19.		miR-223	Glut4, HDAC4, Pknox1, Nfat5	Down	(34)
20.		miR-335	Mest	Up	(47)
21.		miR-107	CAV-1	Up	(47)
22.		miR-223	STAT3	Up	(46)
23.		miR-143	ORP8, AKT	Up	(48)
24.		miR-935	CNR1, ESR1	Up	(46)
25.	Diabetic retinopathy	miR-146a	Fibronectin	Down	(49)
26.		miR-200b	VEGF	Down	(50)
27.		miR-29b	RAX	Up	(40)
28.		miR-195	SIRT1	Up	(51)
29.		miR-486	p53	Up	(52)
30.	Diabetic nephropathy	miR-192	SIP1, ZEB1/ZEB2	Up	(53–55)
31.		miR-21	PTEN, PI3K, Akt	Up	(56, 57)
32.		miR-377	PAK/SOD	Up	(58)
33.		miR-216a	PTEN, Ybx1	Up	(59, 60)
34.		miR-217	PTEN	Up	(60)
35.		miR-93	VEGF	Up	(61)
36.		miR-146a		Up	(62)
37.		miR-155		Up	(63)
38.		miR-25	NOX-4	Down	(64)
39.		miR-215	ZEB2	Down	(55)
40.		miR-29a/b/c	Col1, Col4	Down	(65)
41.		miR-135	TRPC1	Up	(66)
42.		miR-150-5p		Up	(41)
43.		miR-362-3p		Up	(41)
44.		miR-877-3p		Up	(41)
45.		miR-15-5p		Down	(41)
46.	Diabetic cardiovascular disease	miR-16	Cox-2	Down	(67)
47.		miR-133	RhoA, Cdc42	Down	(68)
48.		miR-223	GLUT4	Up	(69)
49.		miR-492	Resistin	Down	(70)
50.		miR-320	IGF-1	Up	(24)
51.		miR-503	Ccne1, Cdc25A	Up	(25)
52.		miR-373	Mef2C	Down	(71)
53.		miR-1	Pim-1	Up	(72)
54.		miR-504	Grb10, Egr2	Up	(73)
55.		miR-24		Down	(74)
56.	Diabetic neuropathy	miR-184-5p			(75)
57.		miR-190a-5p			(75)
58.		miR-182	NOX-4	Up	(23)
59.		miR-146a		Up	(76)
60.		miR-29b	Smad3	Down	(77)

## Proteins as Specific Marker for T2DM

**Figure 1:**
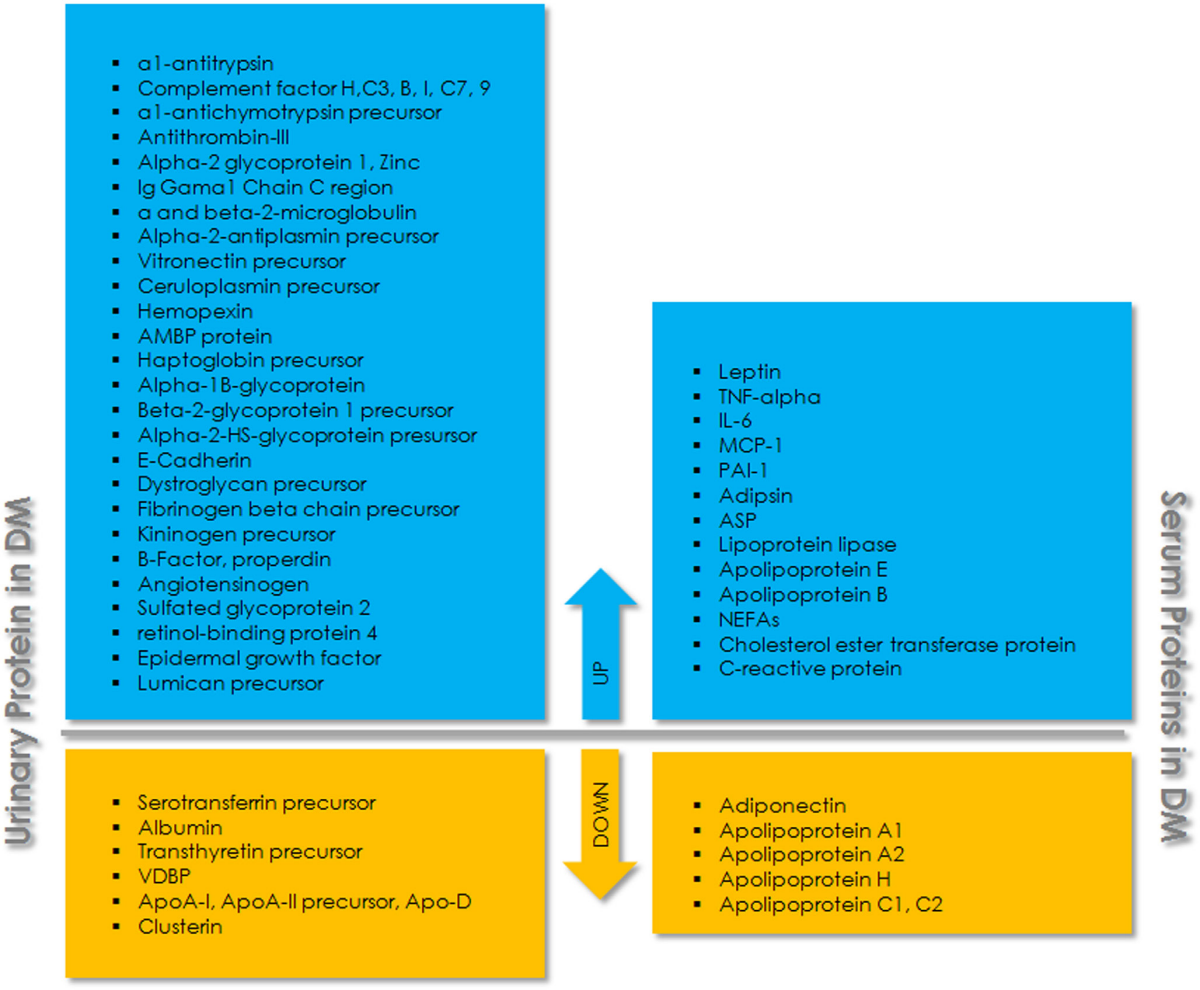
Schematic overview of urinary and serum proteins associated with type 2 diabetes mellitus (T2DM). The upper panel (blue) indicates upregulated proteins and the lower panel (yellow) indicates downregulated proteins in urine and serum.

Proteomic analysis of serum, plasma, and other body fluids using 2D-liquid chromatography and mass spectrometric analysis identified many proteins as biomarkers for T2DM and related complications (Figure 1). Fat accumulation in human body before the onset of T2DM promotes the release of adipokines from adipocytes including adiponectin, leptin, glycoalbumin, and retinol-binding protein 4 (RBP4). Among these, adiponectin and leptin are relatively common and are occasionally used as biomarker for diabetes screening. Glycated albumin is also expressed as some percentage of serum albumin and is involved in only short-term glycemic control (78). RBP4 is another adipocyte-derived factor, reported to be involved in the onset of adiposity and insulin resistance. It is mainly produced in the liver and acts on muscle and/or liver *via* mechanisms that are either retinol-dependent or independent (79). Yang et al. (2005) (80) have shown that the expression of serum RBP4 was higher in insulin-resistant mice and humans with obesity and T2DM. The overexpression of RBP4 in wild-type mice causes insulin resistance while genetic depletion of *Rbp4* improves insulin sensitivity, suggesting that the depleting level of RBP4 could be helpful in the treatment of T2DM, and levels of RBP4 could serve as a biomarker for T2DM. Proteomic study to assess the association of plasma proteins with the risk of developing T2DM has shown that RBP4 is independently associated with the risk of developing T2DM (81). Similarly, low level of adiponectin was found to be associated with an increased risk of development and progression of T2D in different populations. Candidates with increasing adiponectin had a reduced risk of developing T2D (*p* < 0.001) (82, 83). Further, the proteomic analysis of vitreous in diabetic retinopathy has identified six proteins, including pigment epithelium-derived factor, ApoA-1/4, thyroid hormone receptor interactor II, RBP4, and vitamin D binding, as specific marker for diabetic while control had only Apo-H (84). Festa et al. 2002 (85) have shown the significant relation of C-reactive protein (CRP), fibrinogen, and PAI-1 to the development of T2DM. Serum protein profiles of normal and streptozotocin-induced diabetic rat have identified eight proteins with an increased expression in diabetes (86). One such protein, CRP, was found to be associated with inflammation, the progression of disease, and an increased cardiovascular risk in patients (87). Also, in comparison to healthy individuals, individuals with insulin resistance and T2DM have shown difference in the level of many proteins including interleukin-6, resistin, leptin, adiponectin, and visfatin (88–92). A panel of 64 circulating candidate biomarkers was analyzed to develop a model for the assessment of a 5-year risk of developing T2DM and identified six biomarkers including adiponectin, CRP, ferritin, interleukin-2 receptor A, and insulin that provide a better estimation of the risk of developing T2DM than that of FPG levels alone (93). Earliest marker for DN includes excreted albumin in urine. However, further studies revealed that albuminuria is not a suitable marker to assess DN. It has been found that most of the diabetic individuals with the progression of renal disease are normoalbuminuric. Also, albuminuric patients with T2DM have shown biopsies with normal glomerular structure or non-DKDs (94, 95). This suggests the assessment of DN with albuminuria as biomarker lack specificity and sensitivity. Therefore, more urinary biomarkers were investigated for T2DM complications. Urinary monocyte chemoattractant protein-1 (uMCP-1) and vitamin D-binding protein were found to be significantly elevated in microalbuminuric/macroalbuminuric diabetic patients (96). uMCP-1 and uVDBP levels for the early diagnosis and detection of DN exhibited high sensitivity and specificity. Both of these urinary proteins could be used as potential biomarker for the early detection of DN in T2DM patients. Similarly, E-cadherin levels were found to be elevated ~1.3-fold in T2DM, which further increases to ~5–8-fold with progression to DN (97).

In the initial phase of glucose stimulation, insulin biosynthesis is regulated mainly at the translation level. Previously, it was shown that PDI (protein disulfide isomerase) increases insulin translation by binding to its 5′ UTR in response to glucose (98). Several reports suggest that the levels of PDI increase in response to pathological and physiological conditions causing cellular stress (99). The beta cell stress may lead to an increase in the expression of PDI, resulting in high levels of insulin without circulating glucose. Hence, the amount of PDI or beta cell stress markers may serve as potential biomarker in prediabetes.

Recently, it was also shown that chronic hyperinsulinemia in the absence of sufficient glucose progresses to insulin resistance faster than their corresponding controls of chronic hyperinsulinemia in the presence of sufficient glucose (100). These studies suggest that cellular stress leading to the production of insulin in the absence of glucose stimulation could be a trigger for insulin resistance. Thus, the uptake of glucose at a regular interval or a reduced cellular stress could be one of the therapeutic interventions to arrest the progress of prediabetic to diabetic situation.

## Metabolites as Biomarkers

**Figure 2:**
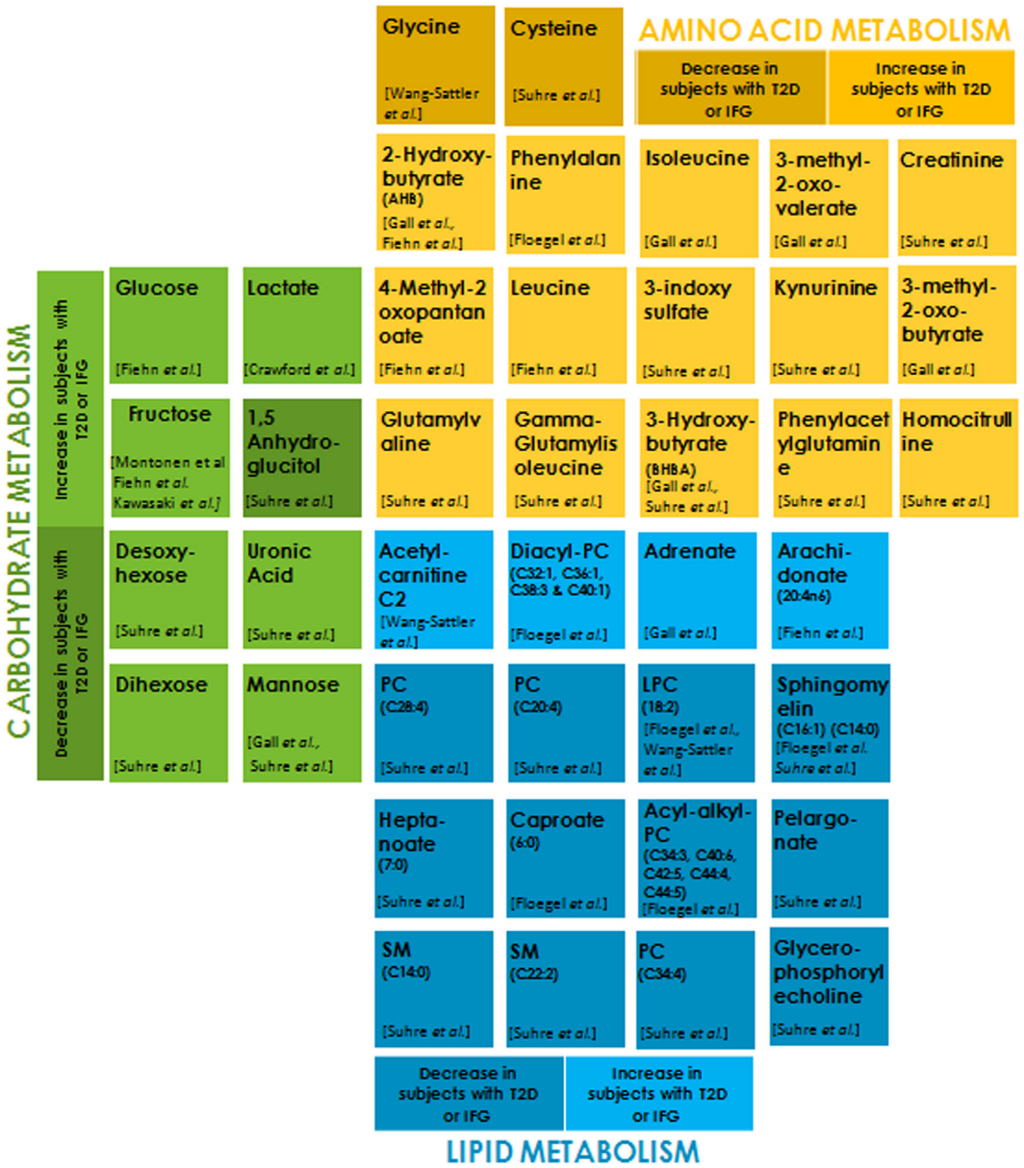
Metabolites belonging to each of the sub groups, i.e., carbohydrate metabolism, amino acid metabolism, and lipid metabolism are differentially color-coded. The light and dark shades of the same color indicate metabolites that are significantly increased and decreased, respectively, in subjects with type 2 diabetes (T2D) or impaired fasting glucose (IFG) with respect to normoglycemic control subjects (*p* < 0.01–0.0001).

Recent advancement in mass spectrometry has made it possible to profile an organism’s metabolic status (101–104). Metabolites are low-molecular-weight compounds that are intermediates or end products of a metabolic pathway. Altered metabolite levels in prediabetic individuals compared to healthy ones may serve as diagnostic biomarkers and enable preventive action (105–110). A large number of metabolites originating from three major fuel sources (carbohydrates, lipids, and proteins) have been implicated as risk factors for the development of T2DM and hence can serve as potential and reliable biomarkers (Figure 2). Serum level of the amino acid glycine is decreased in insulin-resistant individuals. One of the reasons for this could be the increased expression of 5-aminolevulinate synthase 1 (ALAS-H) (111, 112). ALAS-H catalyzes the condensation of glycine and succinyl-CoA into 5-aminolevulinic acid. Glycine is also a substrate for gluconeogenesis, and its reduction indicates increased hepatic gluconeogenesis. Insulin resistance entails a greater insulin secretion as a compensatory mechanism before it becomes eventually exhausted due to β cell dysfunction (113). Phenylalanine is positively correlated to insulin secretion and it may be involved in the early compensatory stage of insulin secretion (114). The most frequent increase in the levels of branched-chain amino acid (BCAA) like valine, leucine (115), isoleucine, and their derivatives 3-methyl-2-oxovalerate and 3-methyl-2-oxobutyrate (116), is attributed to their reduced catabolism. Reduced activities of the key catabolic enzymes, mitochondrial branched-chain amino acid aminotransferase (BCATm) and branched-chain a-ketoacid dehydrogenase (BCKD) in liver and adipose tissue, lead to the accumulation of these amino acids in insulin-resistant individuals (117). Also, poor biotin metabolism is indicative of insulin resistance, leading to impaired BCAA catabolism (118). Gamma-glutamyl derivatives of valine and isoleucine are formed in glutathione-dependent transport of these amino acids. An increase in the circulating concentration of these derivatives may indicate impaired transport of these amino acids in diabetic groups (119). High plasma concentration of α-hydroxybutryrate (2-HB) has been consistently shown to be positively correlated with insulin resistance (115, 116). It has been postulated that this increase in 2-HB results from alteration in methionine/cystathionine catabolic pathways that produces 2-KB (α-ketobutyrate) and cysteine through cystathionine gamma-ligase activity. An increased availability of precursor 2-KB leads to its higher conversion to 2-HB *via* lactate dehydrogenase in insulin-resistant state. β-hydroxybutyrate (or 3-hydroxybutyrate, BHBA) is significantly elevated in diabetes group due to the depletion of hepatic glycogen pool in diabetes, leading to ketogenesis (115, 119). Higher levels of 3 indole-sulfate, creatinine, and homocysteine in diabetic groups may indicate the early onset of impaired renal function that may eventually lead to DN (119). Glomerular filtration rate is a strong determinant of plasma levels of homocysteine and cysteine. Lower plasma levels of cysteine in diabetic patients are indicative of hyperfiltration (119, 120). The type of linkage in the phospholipid core and fatty acid residue plays a key role in determining the T2D risk. Lipids with shorter-chain lengths like pelargonate and heptanoate are depleted in IFG conditions when compared to controls and are associated with T2DM while the long unsaturated fatty acids like adrenate and arachidonate are elevated significantly in T2D patients and may help restore insulin sensitivity. Insulin triggers the expression of various fatty acid desaturase. The above observed changes in the lipid profile may be due to a diminished desaturase activity in insulin-resistant individuals. Also, Rhee et al. (2011) have shown that longer chain triacylglycerol (TAGs) are associated with a decreased risk of diabetes, whereas the short chain TAGs are associated with an increased risk, their levels positively correlate with insulin resistance (121). This may suggest impaired triglyceride lipolysis due to dysregulated glucose metabolism. A higher transcriptional level of carnitine-O-acetyl transferase due to the activation of PPAR–alpha pathway in peroxisome (122) produces acetylcarnitine from carnitine and acetyl-CoA in the mitochondrial matrix. This eventually results in elevated levels of acetylcarnitine C2 in IGT individuals (Figure 2).

Accumulating evidences indicate a positive correlation between serum glucose in T2DM and IGT groups compared to normal glucose tolerance (NGT) controls (115, 123). Insulin resistance and insufficient secretion lead to weakened glycolysis and muscle glycogen synthesis, resulting in the obvious accumulation of circulating glucose in the blood. Lactate is elevated in obese, insulin-resistant subjects and can serve as an independent risk factor for the development of T2DM (124–126). A community-based study on obesity and T2DM showed that a decreased oxidative capacity in these individuals leads to higher plasma levels of lactate (127). The serum and urinary levels of fructose are significantly increased in patients with diabetes due to impaired fructose metabolism in hyperglycemia (115, 128, 129). Elevated levels of modified forms of glucose like desoxyhexose (primarily deoxyglucose), uronic acid (primarily glucuronic acid), dihexose (primarily maltose) (119), and mannose (116, 119) in diabetes group reflect an increased availability of glucose, resulting in their biosynthesis. Reduced levels of 1,5 anhydroglucitol (1,5 AG), a deoxy form of glucose, serve as short-term glycemia marker. A decrease in plasma concentrations of 1,5 AG levels in diabetes as compared to the control results from its renal loss stimulated in hyperglycemic conditions by glycosuria (119).

## Concluding Remark and Future Perspectives

MicroRNA-based prognosis, diagnosis, and disease management propose an exciting idea in the context of T2DM. The expression level of miRNAs not only offers an assessment of pathological state of disease but in some cases could also be used as a therapeutics target. For instance, silencing of miR-103 and miR-107 significantly reduces hyperglycemia in murine model of obesity and T2DM by promoting insulin signaling in liver and adipose tissue (130). Even if the level of many miRNAs promisingly offers an idea about the pathophysiology of T2DM, many issues are yet to be solved before using them in therapeutics or as predictive biomarkers. The development of processing procedure of the small biomolecules from body fluids, their storage conditions, and defined sample preparation is still a major issue that needs to be resolved. Most of these identified biomarkers have varying protocols for plasma preparation. Besides, these procedures can alter their level in the final sample. Thus, an optimized protocol for sample preparation needs to be developed for these biomarkers. The physiological issue associated with biomarkers like miRNAs is their implication in regulating multiple molecular pathways. Effect on multiple pathways is needed to be addressed to assess their specificity and accuracy as well as identifying the interconnectivity of various networks.

Most methods for the screening and prevention of T2DM rely on prediabetes individuals already showing a steady decrease of insulin sensitivity. However, these methods may not be as effective as those developed to counter the disease even before the onset of this stage. Hence, it is important to develop biomarker trajectory models that can complement accurately with the existing individual risk assessment methods. For example, the diagnostic potential of 1-h plasma glucose (1-h PG) of ≥155 mg/dl is better than the current threshold levels of FPG, 2-h PG, or HbAc1 for prediabetes, identifying high-risk individuals at the so-called pre-prediabetes stage (131). This is because the beta cell function is substantially intact at this stage, and hence lifestyle interventions might be more effective in potentially reducing progression to diabetes (132).

The major shortcoming of multimarker approach is the fact that the overlap of biomarker concentrations between individuals with and without the incidence of T2DM is significant, compromising its discriminative ability. Most novel circulating and/or genetic biomarkers show a high degree of correlation with the existing risk factors adding little or no value. Risk models containing measures of biomarkers belonging to the same casual pathway as that of the disease itself may not improve the predictability of the disease.

## Outstanding Questions

How do these identified signature molecules like miRNA apply as biomarkers in the context of larger population with genetic variations? Owing to their association with multiple targets, the specificity of miRNA is an issue. Will profiling of miRNAs in body fluids and proteins at different pathophysiological stages of disease using larger study groups help in the selection of potential candidates among identified biomarkers? Further research is needed to understand the mechanism of regulation of these biomolecules and how their altered levels in body fluids specifically relate to T2DM.

## Author Contributions

The primary manuscript was prepared by SV and RS with idea input from VS.

## Conflict of Interest Statement

The authors declare that the research was conducted in the absence of any commercial or financial relationships that could be construed as a potential conflict of interest.
